# Language of Anxiety on Social Media: Distinguishing “Anxious” from “Nervous” in Youth Conversations

**DOI:** 10.1192/j.eurpsy.2025.1167

**Published:** 2025-08-26

**Authors:** C. Noureddine, A. Sterchele, C. Pham, C. Wang

**Affiliations:** 1 Psychiatry, Icahn School of Medicine at Mount Sinai, New York, United States

## Abstract

**Introduction:**

Social media has evolved into a primary arena for mental health discussions, with particular relevance to youth who engage extensively in online spaces. This study seeks to investigate how distinct language choices—specifically the terms “anxious” and “nervous”—influence the tone, themes, and context of discussions around anxiety.

**Objectives:**

By examining the frequency and context of these terms in social media posts, this study aims to shed light on how language affects the portrayal of anxiety and, potentially, the associated experiences. We hypothesize that understanding these differences can provide valuable insights into public perceptions especially with younger audiences.

**Methods:**

We conducted a targeted search of X (formerly Twitter), focusing on posts that included either the term “anxious” or “nervous,” filtered for English-language content to ensure relevance to a broader audience. The top 100 tweets containing each term were then subjected to a sentiment analysis to assess the emotional tone of the content. Additionally, a word cloud generator was used to identify the most frequently associated terms, with connecting words excluded to maintain clarity in theme identification.

**Results:**

Posts containing the term “anxious” often reflected intense personal distress, introspection, and struggles with internal emotions. The most commonly associated terms included “feel” (mentioned 19 times), “god” (16), “like” (15), “can” (14), “don’t” (14), and “stressed” (8), each of which underscored the personal and often existential nature of these expressions. The frequent presence of terms such as “always,” “day,” “got,” “walk,” “hate,” and “sad” further emphasized the internalized and enduring aspects of anxiety, suggesting more persistent or generalized anxiety concerns.

On the other hand, posts containing the term “nervous” were more likely to relate to situational anxiety, external circumstances, or performance-based fears. The most frequently appearing words alongside “nervous” included “system” (15 mentions), “can” (13), “just” (9), “like” (8), and “people” (7), indicating that these posts often referenced responses to specific events or temporary stressors. Notably, terms like “first,” “lot,” “making,” “now,” and “video” suggest that individuals using “nervous” may be describing anxiety linked to immediate, often identifiable stressors, as opposed to the more generalized or chronic anxiety depicted in “anxious” posts.

**Conclusions:**

This study underscores the significance of linguistic nuances in mental health conversations on social media and highlights how the choice of words—such as “anxious” versus “nervous”—can shift both the perceived and intended meaning of these discussions. By understanding these subtle differences, mental health professionals, educators, and content creators can develop resources and messaging that are more attuned to the experiences and language of youth.

**Disclosure of Interest:**

None Declared

